# Comprehensive Analysis and Comparison on the Codon Usage Pattern of Whole* Mycobacterium tuberculosis* Coding Genome from Different Area

**DOI:** 10.1155/2018/3574976

**Published:** 2018-05-08

**Authors:** Li Gun, Ren Yumiao, Pan Haixian, Zhang Liang

**Affiliations:** Department of Biomedical Engineering, School of Electronic Information Engineering, Xi'an Technological University, Xi'an, China

## Abstract

Phenomenon of unequal use of synonymous codons in* Mycobacterium tuberculosis* is common. Codon usage bias not only plays an important regulatory role at the level of gene expression, but also helps in improving the accuracy and efficiency of translation. Meanwhile, codon usage pattern of* Mycobacterium tuberculosis* genome is important for interpreting evolutionary characteristics in species. In order to investigate the codon usage pattern of the* Mycobacterium tuberculosis* genome, 12* Mycobacterium tuberculosis* genomes from different* area* are downloaded from the GeneBank. The correlations between G_3_, GC_12_, whole GC content, codon adaptation index, codon bias index, and so on of* Mycobacterium tuberculosis* genomes are calculated. The ENC-plot, relationship between A_3_/(A_3_ + T_3_) and G_3_/(G_3_ + C_3_), GC_12_ versus GC_3_ plot, and the RSCU of overall/separated genomes all show that the codon usage bias exists in all 12* Mycobacterium tuberculosis* genomes. Lastly, relationship between CBI and the equalization of ENC shows a strong negative correlation between them. The relationship between protein length and GC content (GC_3_ and GC_12_) shows that more obvious differences in the GC content may be in shorter protein. These results show that codon usage bias existing in the* Mycobacterium tuberculosis *genomes could be used for further study on their evolutionary phenomenon.

## 1. Introduction

Tuberculosis (TB) is caused by* Mycobacterium tuberculosis *that most often affect the lungs. Today, TB is still an unsolved problem. According to the WHO, in Oct, 2017, fact sheet pointed out that the TB is one of the top 10 causes of death worldwide [1]. It spreads from person to person through the air. One person may be infected by TB when only inhaling a few of TB germs. So, many scientists studied the characteristics including codon usage pattern [2], promoter architecture of toxin-antitoxin systems [3], drug-resistance [4], molecular epidemiology [5], protein function and immunogenicity [6], and drugs for the treatment [7] of TB. Typically, Sheen et al. studied the multiple genomes of* Mycobacterium tuberculosis* and found out some specific novel genes and mutations associated with pyrazinamide resistance [8]. Sun et al. and Khrustalev et al. characterize the mutations in streptomycin-resistant* Mycobacterium tuberculosis* [9, 10]. Recently, bioinformatics tools are widely used for analyzing the TB [11].

One amino acid is often encoded by more than one codon because the genetic code is redundant. This phenomenon is also known as synonymous codon usage. Many factors may affect the codon usage in* Mycobacterium tuberculosis*, such as mutation pressure [12], gene length [13], and natural selection [14]. Most previous studies deal with the relatively smaller set of* Mycobacterium tuberculosis *isolates [15, 16] or the isolates from one area [17, 18]. In this paper, genetic diversity of* Mycobacterium tuberculosis* from 12 areas is compared and analyzed. So, the objectives of this study aim to deal with the following issues: (1) correlation between codon preference parameters such as the codon usage index (CBI), the effective number of codons (ENC), the overall GC content, the codon adaptation index (CAI), and the Frequency of Optimal Codons (FOP); (2) mutation and evolution characteristics via the relationship between ENC and the GC_3_ of the whole 12* Mycobacterium tuberculosis* genomes; (3) phylogenetic analysis via RSCU values of the separated genomes for* Mycobacterium tuberculosis*.

## 2. Materials and Methods

The sequences of* Mycobacterium tuberculosis* (accession numbers in NCBI are shown in the Table 1) from Peru, Argentina, China, and so on in Homo sapiens are downloaded and examined. Specifically, coding sequences within* Mycobacterium tuberculosis* genome are selected according to the following: (1) containing more than 300 bases, (2) starting with the start codon, (3) number of bases being a multiple of 3, and (4) having no stop codon in the inner sequence. Thus, a total of 12965 coding sequences for* Mycobacterium tuberculosis* are selected (originally the total of coding sequences is 24520).

### 2.1. Effective Number of Codons (ENC) Analysis

ENC analysis can be used to quantify the absolute codon usage bias in coding sequences. ENC was calculated using the following formula [19]:

(1) $$\begin{array}{c} ENC^{calculatied}=2+\frac{9}{{\overset{-}{f}}_{2}}+\frac{1}{{\overset{-}{f}}_{3}}+\frac{5}{{\overset{-}{f}}_{4}}+\frac{3}{{\overset{-}{f}}_{6}}, \end{array}$$

where ${\overset{-}{f}}_{k}$  (*k* = 2,3, 4,6) is the mean of *f*_*k*_ values for the *k*-fold degenerate amino acids; ${\overset{-}{f}}_{k}$ denotes the average homozygosity for the amino acid class whose degree of codon degeneracy is *k*. The coefficients 9, 1, 5, and 3 denote the number of amino acids belonging to different classes. Here, *f*_*k*_ is estimated using the formula: *f*_*k*_ = (*nS* − 1)/(*n* − 1), where *n* is the total number of occurrences of the codons for that amino acid and *S* = ∑(*n*_*i*_/*n*)^2^  (*i* = 1 ⋯ *k*), and *n*_*i*_ here is the total number of occurrences of the *i*th codon for that amino acid. Genes for which the codon choice is only constrained by a mutation bias will lie on or just below the curve of the expected ENC values [20]. Therefore, to elucidate the relationship between GC_3_ and ENC values, the expected ENC values for different GC_3_ were calculated as follows [21]:

(2) $$\begin{array}{c} ENC^{expectation}=2+s+\frac{29}{s^{2}+{\left(1-s\right)}^{2}}, \end{array}$$

where *s* represents the given GC_3_%.

### 2.2. RSCU Value Analysis

The RSCU values for all genes in* Mycobacterium tuberculosis* are calculated to determine the characteristics of synonymous codon usage without the confounding influence of amino acid composition and coding sequence size of different gene samples following a previously described method [22]. The RSCU index was calculated as follows:

(3) $$\begin{array}{c} RSCU=\frac{g_{ij}}{\sum_{j}^{n_{i}}g_{ij}}n_{i}, \end{array}$$

where *g*_*ij*_ is the observed number of the *i*th codon for the* j*th amino acid, which has *n*_*i*_ kinds of synonymous codons. RSCU values represent the ratio between the observed usage frequency of one codon in a gene sample and the expected usage frequency in the synonymous codon family, given that all codons for the particular amino acid are used equally. The synonymous codons with RSCU values > 1.0 have positive codon usage bias and were defined as abundant codons, whereas those with RSCU values < 1.0 have negative codon usage bias and were defined as less-abundant codons.

### 2.3. Codon Adaptation Index (CAI) Calculation

The CAI value is generally used to predict the expression level of an inbred gene (it is more suitable for unicellular organisms) and can be used to predict the expression level of foreign genes. The CAI value can be calculated via the following equation [23]:

(4) $$\begin{array}{c} CAI={\left(\;\prod_{K=1}^{L}w_{K}\;\right)}^{1/L}, \end{array}$$

where *L* refers to the number of codons used in the gene. The value of CAI is between 0 and 1; the larger the value, the stronger the codon usage bias. *w*_*ij*_ is the relative adaptiveness of a codon, which can be calculated via

(5) $$\begin{array}{c} w_{ij}=\frac{{RSCU}_{ij}}{{RSCU}_{imax}}. \end{array}$$

In (5), the RSCU_*ij*_ denotes RSCU value of the codon for the *i*th amino acid. And the RSCU_*i*max_ is the RSCU value for the most frequently used codon for the specific amino acid. The value of the CAI falls between 0 and 1; larger value denotes the larger bias of the codon usage.

### 2.4. Calculation for the Codon Bias Index (CBI)

The codon bias index reflects the presence of components with high codon usage in a particular gene. The value of CBI has been widely used for it can more clearly describe the foreign gene expression in the host. The CBI calculation method can be expressed by the following formula [24]:

(6) $$\begin{array}{c} CBI=\frac{N_{opt}-N_{ran}}{N_{tot}-N_{ran}}, \end{array}$$

where the *N*_opt_ represents the total number of occurrences of the superior codon in the gene; in this work, the superior codons are the codons whose RSCU value is more than 1.6. *N*_ran_ represents the sum of the number of occurrences of the superior codon when all the synonymous codons are random in a certain protein; *N*_tot_ represents the occurrence number of the amino acid corresponding to the superior codon in the gene. In this work, the CBI of genes for* Mycobacterium tuberculosis* genome is calculated and analyzed. Accordingly, the Frequency of Optimal Codons (FOP) of* Mycobacterium tuberculosis* genome, which is the weighted average of the RSCU of superior codons (RSCU value > 1.6), is calculated similarly.

### 2.5. Parity Rule 2 (PR2) Analysis

The parity rule 2 (PR2) plot is usually used to estimate the impact of mutation and selection pressure on codon usage of genes. In the PR2 plot, the abscissa is [G_3_/(G_3_ + C_3_)] denoting the GC-bias at the third codon position in entire coding sequences, while the ordinate is the A_3_/(A_3_ + U_3_) which denotes the AU-bias at the third codon position of entire coding sequences [24]. In this work, the effects of mutation pressure and natural selection on the codon usage of genes in* Mycobacterium tuberculosis* will be analyzed via the PR2 analysis.

### 2.6. Neutral Evolution and Clustering Analysis

The neutrality plot is the so-called neutral evolution analysis, which could be performed to determine and compare the extent of influence of mutation pressure and natural selection. In this work, the neutral evolution analysis of the codon usage pattern of* Mycobacterium tuberculosis* is conducted and analyzed by plotting the GC_12_ values of the synonymous codons against the GC_3_ values. In the present study, clustering analysis was used to analyze the difference in codon usage patterns among* Mycobacterium tuberculosis* via RSCU of coding sequences. In the clustering analysis progress, in order to minimize the effect of amino acid composition on codon usage, the three stop codons and two particular amino acid codons, AUG and UGG, are excluded. The pdist and dendrogram function of Matlab are used to build the tree. The function could generate a dendrogram plot of the hierarchical binary cluster tree. Others, such as the relationship between ENC and protein length, relationship between CBI and protein length, relationship between overall GC content and protein length, histogram of CBI, FOP, and overall GC content, as well as the protein versus GC_12_/GC_3s_ are all explored.

## 3. Results and Discussion

Codon usage pattern belonging to compositional characteristics such as the T_3_, C_3_, A_3_, G_3_, GC_12_, and GC_3_ and overall GC content of 12* Mycobacterium tuberculosis* genomes from different area are calculated. The results show that the average value of overall GC content is 65.36%, average value of GC_3_ is 79.18%, and the average T_3_ (11.38%) and A_3_ (9.43%) of 12* Mycobacterium tuberculosis* genomes are fewer than GC_3_. Furthermore, the average ENC, FOP, CBI, and CAI are 41.25, 0.64, 0.39, and 0.45.

**Figure 1:**
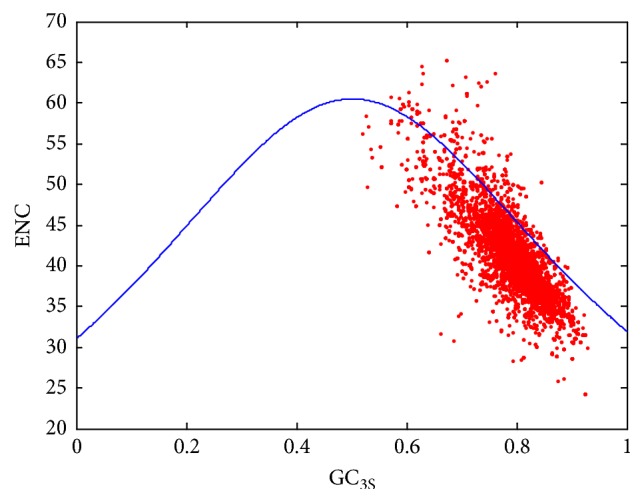
Plot of ENC versus GC_3_ within* Mycobacterium tuberculosis* genomes.

ENC is an important index to measure the codon usage bias in a genome and play a major role in their codon usage profile. In order to investigate the synonymous codon usage pattern of the* Mycobacterium tuberculosis* genomes, the ENC versus GC_3_ is plotted and the result is shown in Figure 1. Every point represents one coding gene in* Mycobacterium tuberculosis*. It can be seen that most dots are under the expected curve. Many scientists tended to think that the ENC value is belonging to a range of 21 to 61, but when the *f*_*k*_ is calculated via (*n*∑(*n*_*i*_/*n*)^2^ − 1)/(*n* − 1), all ENC values fell in the range from 20 to infinite; here, the value 20 denotes the codon usage bias using only one possible synonymous codon to correspond to an amino acid, and the larger value indicates that there is less bias of using all possible synonymous codons, and the frequency of using all possible synonymous codons may tend to be equal.

**Figure 2:**
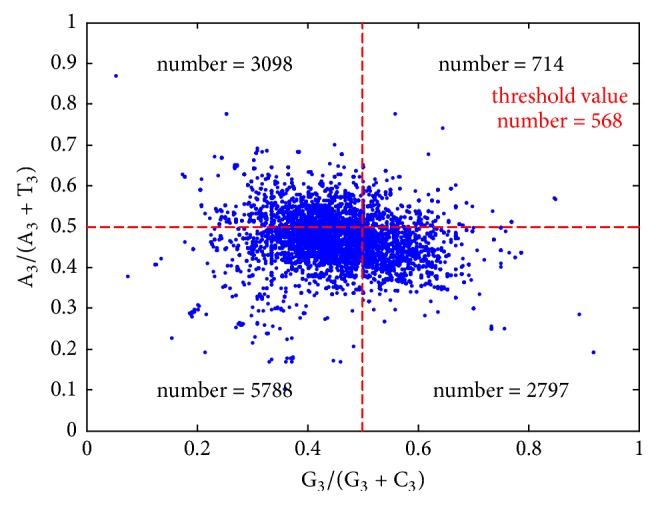
PR2-bias plot analysis of* Mycobacterium tuberculosis* genomes.

The parity rule states that if there is no mutation in genes, or no bias on the codon selection effect, the base content should obey the laws A = T and G = C. This method is usually used to analyze the PR2 bias of the third place codon via comparing the A_3_/(A_3_ + T_3_) and the values of G_3_/(G_3_ + C_3_). The PR2 bias plot of 12* Mycobacterium tuberculosis* genomes is shown in Figure 2. The distance between dot and the center denotes the degree and direction of the PR2 bias. From Figure 2, in most genes, A_3_ are less than T_3_. Most C_3_ content of the genes are more than the occurrence rate of T3 in these genes.

**Figure 3:**
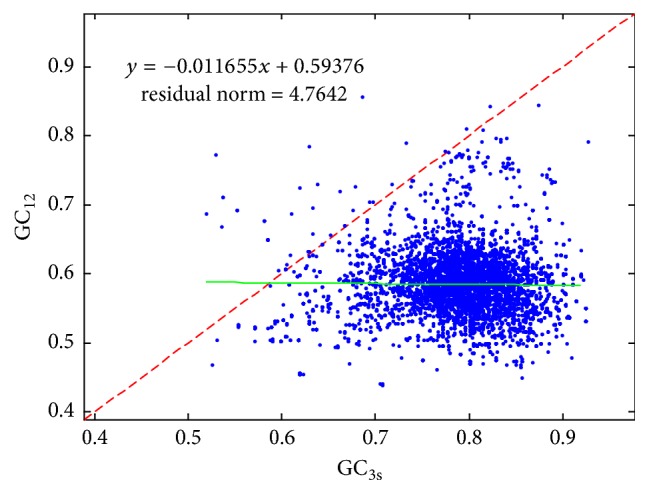
Neutrality plot of GC12 versus GC3s for 12* Mycobacterium tuberculosis* genomes.

Usually, the neutrality plot is used to analyze the directional mutation pressure versus natural selection of a certain genome, which could also reveal the relationship between the GC_12_ and the GC_3_ with the GC12 as the ordinate and the GC_3_ as the abscissa. The neutrality plot of 12* Mycobacterium tuberculosis* genomes is shown in Figure 3. Each point shown in Figure 3 represents one separate gene for the* Mycobacterium tuberculosis*. The result shows that the distribution of most GC_12_ rate is between 0.5 and 0.65 and most of the GC_3_ rate is during 70%–90%. In addition, GC_3_ is more than GC_12_ in most of the genes. Meanwhile, the correlation analysis in Table 2 shows that there is a weak correlation with the value of −0.015. Form these data performance, it can be seen that the natural selection may be a greater impact factor on codon preference in* Mycobacterium tuberculosis* genomes.

**Figure 4:**
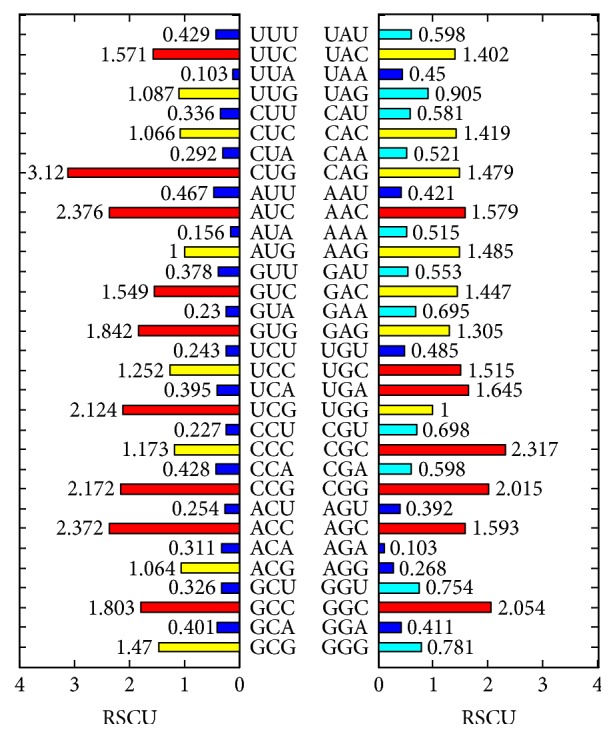
Overall RSCU of the 12* Mycobacterium tuberculosis* genomes.

RSCU value, which is an important parameter for evaluating the bias of the synonymous codon, represents the ratio between the occurrence frequency of one codon and the expected usage frequency in a gene sample. Codons whose RSCU values are more than 1.0 would be regarded as having positive codon usage bias. On the contrary, those RSCU values less than 1.0 are defined as the less-abundant codons. The overall RSCU value can intuitively reflect the preference for codon usage in a certain genome. The overall RSCU values of 12* Mycobacterium tuberculosis* genomes are calculated and the results are shown in Figure 4. The codon usage bias shows that the red bars in those RSCU values are more than 1.5, which could be regarded as the abundant codons; these codons are UUC, CUG, AUC, GUC, GUG, UCG, CCG, ACC, AAC, UGC, UGA, CGC, CGG, AGC, and GGC. Among them, CUG has the highest RSCU value of 3.12. The blue bars in Figure 4 are showing the opposite (RSCU values are less than 0.5) characteristics which could be called the less-abundant codons. Generally, RSCU values for the codons ending with the A or T are smaller than the RSCU value of the codons ending with G or C. There are three stop codons (UGA, UAA, and UAG), and no corresponding amino acid corresponding to stop codons. In Figure 4, RSCU values for terminal codons are UGA = 1.645, UAA = 0.45, and UAG = 0.905, and sum of them is 3 (there are 3 stop codons). On the other hand, the RSCU value 1.645 for UGA here denotes that 54.8% (1.645/3) of stop codons are UGA, which is more than the sum of UAA and UAG.

**Figure 5:**
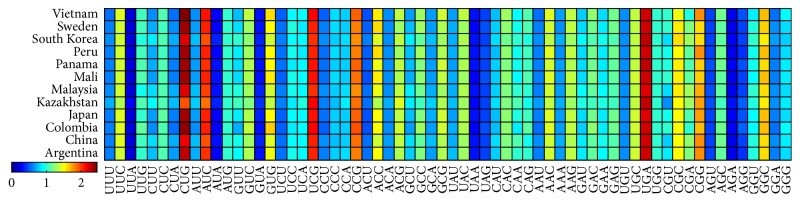
Heatmap of RSCU values for 12 separated* Mycobacterium tuberculosis* genomes.

The value of RSCU is usually used as a measurement of the codon usage bias. The overall RSCU values (Figure 4), to a certain degree, can reflect the characteristics of codon usage, but when it comes to the codon usage of individual sequence for a particular genome, it can be seen that there is a great difference of RSCU values from different genes even of one genome. RSCU values of 12* Mycobacterium tuberculosis* genomes are separately shown in Figure 5. There is some difference between all genomes especially in the usage of CUU, GUG, ACC, GAU, CGG, GGC, and so on.

The CBI value can more clearly describe the foreign gene expression in the host. The CBI values of 12 separated* Mycobacterium tuberculosis* genomes are calculated via (6). Meanwhile, in order to explore the relationship between the CBI and the ENC, the ENC values are equalized to the range of the CBI via the following equation:

(7) E.ENC=ENC−ENCminENCmax−ENCminCBImax−CBImin+CBImin.

**Figure 6:**
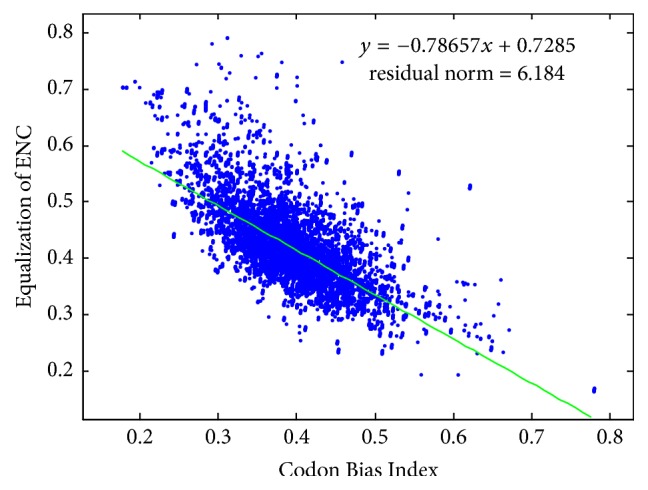
Overall relationship between CBI and the equalization of ENC.

Most previous researchers pointed out that the smaller ENC value may denote higher codon usage bias (CBI). The phenomenon is shown in Figure 6, and here the negative relationship between them is digitized by the linear fitting results. The results show that the negative correlation coefficient is −0.78657.

**Figure 7:**
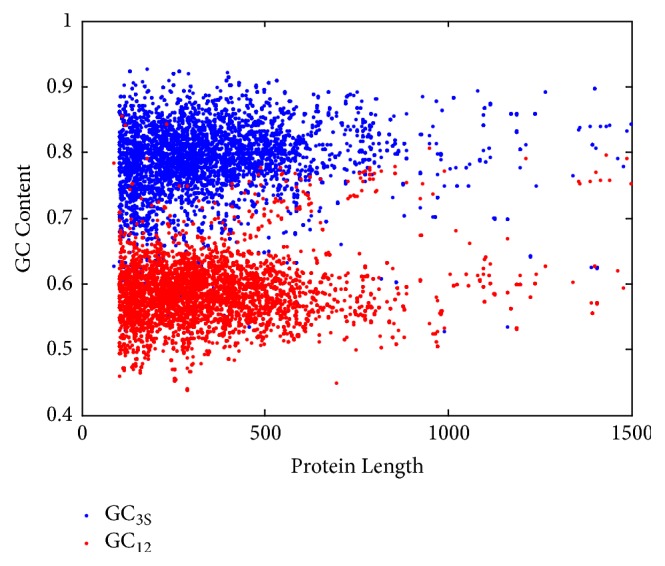
Relationship between protein length and GC content (GC_3S_ and GC_12_).

Proteins in* Mycobacterium tuberculosis *could be analyzed via many methods, mass spectrometry, codon usage, and so on [25, 26]. Codon usage is linked to nucleic acids and proteins, especially the GC content. In Figure 7, relationship between protein length and GC content (GC_3_ and GC_12_) is described. Most proteins are concentrated within five hundred amino acids in length; it can also be seen from Figure 7 that when encoding the same length of protein, most of the GC_3_ content is higher than the GC_12_ content. As the length of the code increases, the GC content of either GC_12_ or GC_3_ tends to increase slightly. But within the lengths between 1000 and 2000 nucleotide residues, the GC-contents (GC_12_ and GC_3_) are not obviously varied with the gene length (Okazaki fragment in bacteria is between 1000 and 2000 nucleotide residues; its corresponding protein length is between 333 and 667 or so). Here, only proteins of less than 1500 amino acids in length are considered.

**Figure 8:**
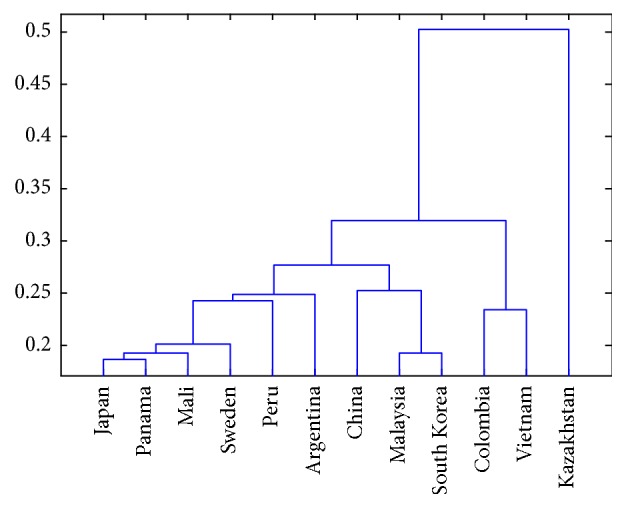
Phylogenetic tree of 12* Mycobacterium tuberculosis *genomes.

Codon bias has a very broad significance for exploring a genome. From the molecular level, codon bias can also explain the basic phenomenon of evolutionary process in biology [27]. Sometimes, the differences between RSCU values of different genomes can be used to describe the evolutionary distance. Numerical closeness of RSCU values between coding sequences shows the closeness of relationship [28]. Genetic diversity of* Mycobacterium tuberculosis *from different area may have different frequency of ancestral strains [29]. Diversity and disease pathogenesis for* Mycobacterium tuberculosis *from different area may show difference too [30, 31]. When the evolutionary relationship of 12 separated* Mycobacterium tuberculosis* is concerned, samples from different countries have subtle differences. Genetic relationship of them is shown in Figure 8.

*Mycobacterium tuberculosis* exists in most countries and exploring its codon usage bias is useful for understanding genetic characteristics and geographical differences of whole genomes. Although many scientists endeavor to study the* Mycobacterium tuberculosis* from the genetic perspective [32, 33] for its very important for defending* tuberculosis* [34], its codon usage bias is not very clear until now. In this study, not only are traditional methods, such as ENC-plot, PR2 analysis, RSCU values, and phylogenetic analysis of 12* Mycobacterium tuberculosis *genomes, studied, but also some new methods, CBI versus ENC and protein length versus GC_3S_/GC_12_, are all explored; the former relationship describing the relationship between CBI and ENC could reveal their negative correlation characteristics. Relationship between protein length and GC content (GC_3S_ and GC_12_) can reflect the changes of GC content within different coding sequences during the evolutionary process. Previous studies indicated that genetic diversity exists in* Mycobacterium tuberculosis* isolated from different* tuberculosis* [35] when the samples are from different areas. Many studies also revealed that the host is an important factor that can affect the codon usage characteristics [36]. Overall RSCU values of overall 12* Mycobacterium tuberculosis* genomes (Figure 4) and their separated genomes (Figure 5) all reveal strong codon usage bias. All these results combined with the relationship between CBI and ENC, protein length, and GC content (GC_3_ and GC_12_) show that the natural selection pressure is a little more important than mutation pressure. There are only 12 genomes of* Mycobacterium tuberculosis* analyzed in this work; although* Mycobacterium tuberculosis *exists in almost all countries. We selected 12 geographically representative genomes in order to analyze their regional differences. Codon usage differences of* Mycobacterium tuberculosis *from broader range and even from one area are not mentioned in this work; it is necessary to expand the scope of the genomes in the further study.

## 4. Conclusions

In this paper, the codon usage patterns of 12* Mycobacterium tuberculosis *genomes, such as the ENC-plot, the A_3_/(A_3_ + T_3_) versus G_3_/(G_3_ + C_3_) plot, the relationship GC_12_ versus GC_3_, the RSCU of overall/separated genomes, the relationship between CBI and the equalization of ENC, and the relationship between protein length and GC content (GC_3S_ and GC_12_), and their phylogenetic relationship are all analyzed. The codon usage pattern and its influencing factors, especially, were identified for 12* Mycobacterium tuberculosis* genomes. It is observed that codon usage patterns in* Mycobacterium tuberculosis* genomes are influenced by GC3 bias. Correlation between codon bias index and GC3 (=0.618) shows that the GC3 bias of* Mycobacterium tuberculosis* genomes may also reflect its codon bias index. Majorities of the codons are G/C ended; this phenomenon combined with a negative correlation between overall GC-content and CAI may indicate the important role of compositional constraints and mutation pressure in shaping the codon usage bias in* Mycobacterium tuberculosis* genomes. We performed the comparative analysis of codon usage bias in* Mycobacterium tuberculosis* genomes from different area. The results would help further elucidate the underlying dynamics of genetic evolution in* Mycobacterium tuberculosis* genomes. These results showed that codon usage bias exists in the* Mycobacterium tuberculosis *genomes. All this information is important for explaining the function of* Mycobacterium tuberculosis* genomes and helps in understanding the evolutionary process of the* Mycobacterium tuberculosis*.

## Figures and Tables

**Table 1:** Basic characteristics of sequences for* Mycobacterium tuberculosis.*

Accession number	Number of total genes	Number of selected genes	Country
LWDR01000001.1	2048	1166	Argentina
CP011510.1	1973	1138	China
CP017596.1	2062	1154	Colombia
AP017901.1	2141	933	Japan
CP012506.2	2039	1018	Kazakhstan
CP010968.1	1985	1083	Malaysia
JKXT01000001.1	1841	1002	Mali
NZ_CM002052.1	2173	1089	Panama
NZ_CP023622.1	2159	1110	Peru
CP008981.1	1857	1028	South Korea
NZ_CM001226.1	2214	1082	Sweden
AP018033.1	2028	1162	Vietnam

**Table 2:** Correlation of several basic parameters in *Mycobacterium tuberculosis* genomes.

Title	GC	GC_3s_	CAI	CBI	ENC	FoP
GC_3s_	0.536					
CAI	−0.37	−0.588				
CBI	0.263	0.618	−0.325			
ENC	−0.474	−0.809	0.553	−0.685		
FoP	0.28	0.46	−0.134	0.839	−0.612	
GC_12_	0.832	−0.015	−0.056	−0.088	−0.034	0.033
